# A Smartphone Client-Server Teleradiology System for Primary Diagnosis of Acute Stroke

**DOI:** 10.2196/jmir.1732

**Published:** 2011-05-06

**Authors:** J Ross Mitchell, Pranshu Sharma, Jayesh Modi, Mark Simpson, Monroe Thomas, Michael D Hill, Mayank Goyal

**Affiliations:** ^3^Calgary Scientific IncorporatedCalgary, ABCanada; ^2^Department of Clinical NeurosciencesCalgary, ABCanada; ^1^Imaging Informatics LabDepartment of RadiologyUniversity of CalgaryCalgary, ABCanada

**Keywords:** Acute stroke, teleradiology, computed tomography, mhealth, mobile phone

## Abstract

**Background:**

Recent advances in the treatment of acute ischemic stroke have made rapid acquisition, visualization, and interpretation of images a key factor for positive patient outcomes. We have developed a new teleradiology system based on a client-server architecture that enables rapid access to interactive advanced 2-D and 3-D visualization on a current generation smartphone device (Apple iPhone or iPod Touch, or an Android phone) without requiring patient image data to be stored on the device. Instead, a server loads and renders the patient images, then transmits a rendered frame to the remote device.

**Objective:**

Our objective was to determine if a new smartphone client-server teleradiology system is capable of providing accuracies and interpretation times sufficient for diagnosis of acute stroke.

**Methods:**

This was a retrospective study. We obtained 120 recent consecutive noncontrast computed tomography (NCCT) brain scans and 70 computed tomography angiogram (CTA) head scans from the Calgary Stroke Program database. Scans were read by two neuroradiologists, one on a medical diagnostic workstation and an iPod or iPhone (hereafter referred to as an iOS device) and the other only on an iOS device. NCCT brain scans were evaluated for early signs of infarction, which includes early parenchymal ischemic changes and dense vessel sign, and to exclude acute intraparenchymal hemorrhage and stroke mimics. CTA brain scans were evaluated for any intracranial vessel occlusion. The interpretations made on an iOS device were compared with those made at a workstation. The total interpretation times were recorded for both platforms. Interrater agreement was assessed. True positives, true negatives, false positives, and false negatives were obtained, and sensitivity, specificity, and accuracy of detecting the abnormalities on the iOS device were computed.

**Results:**

The sensitivity, specificity, and accuracy of detecting intraparenchymal hemorrhage were 100% using the iOS device with a perfect interrater agreement (kappa = 1). The sensitivity, specificity, and accuracy of detecting acute parenchymal ischemic change were 94.1%, 100%, and 98.09% respectively for reader 1 and 97.05%, 100%, and 99.04% for reader 2 with nearly perfect interrater agreement (kappa = .8). The sensitivity, specificity, and accuracy of detecting dense vessel sign were 100%, 95.4%, and 96.19% respectively for reader 1 and 72.2%, 100%, and 95.23% for reader 2 using the iOS device with a good interrater agreement (kappa = .69). The sensitivity, specificity, and accuracy of detecting vessel occlusion on CT angiography scans were 94.4%, 100%, and 98.46% respectively for both readers using the iOS device, with perfect interrater agreement (kappa = 1). No significant difference (*P* < .05) was noted in the interpretation time between the workstation and iOS device.

**Conclusion:**

The smartphone client-server teleradiology system appears promising and may have the potential to allow urgent management decisions in acute stroke. However, this study was retrospective, involved relatively few patient studies, and only two readers. Generalizing conclusions about its clinical utility, especially in other diagnostic use cases, should not be made until additional studies are performed.

## Introduction

Recent advances in the treatment of acute ischemic stroke have made rapid acquisition, visualization, and interpretation of images a key factor for positive patient outcomes. Some teleradiology systems can accelerate image interpretation and reduce treatment delays [1-3]. However, teleradiology systems have three important features that limit their utility for acute stroke diagnosis. First, the systems may require that patient digital imaging and communications in medicine (DICOM) images be transferred to a remote device for viewing before interpretation can begin. Acute stroke imaging exams often contain several hundred megabytes (MB) of data. The delay caused by transmission of the image data may significantly reduce treatment effectiveness. In addition, if there is confidential information in the headers of the DICOM files, then meeting security requirements may cause additional delays and/or restrict the locations where the remote device may be located. Second, the devices used for remote visualization may limit or inconvenience physician mobility. This may result in additional delays while a physician on call travels to the nearest remote visualization device. Third, mobile devices may lack the computational capabilities to perform advanced visualizations that can aid the diagnostic process. In turn, this may prevent mobile devices from achieving functional equivalence to workstation systems and, therefore, make regulatory approval of mobile devices for this purpose more difficult.

Client-server based teleradiology systems have been described in the literature previously [4,5]. However, these are designed primarily to communicate with workstation class devices. There are no reports in the literature of interactive, streaming, client-server systems that can provide sufficient functionality, image quality, and frame rates to a current generation smartphone device to allow for primary diagnosis.

We have developed a new teleradiology system based on a client-server architecture to try to address these limitations. As a remote visualization device, our system can use an Apple (Apple Inc, Cupertino, CA) iPhone, iPod Touch, iPad (hereafter, referred to as iOS device), or a device running Android (Google Inc, Mountain View, CA) version 2.1 or newer equipped with a touch screen. When using our system, DICOM images are not transferred to the remote device. Instead, the server loads and renders the patient DICOM images, then transmits a rendered frame to the remote device. This process can occur within a few seconds and allows a remote physician to view the first frame and begin interpretation quickly. The server can be placed in a secure location, and all transmissions can occur using standard protocols over secure connections. In addition, since a server is performing the rendering, advanced visualization methods not possible on the remote device, such as three-dimensional volume rendering, may be used to generate frames for remote viewing.

Here, we report results from an initial feasibility study to determine if the new system is capable of providing accuracies and interpretation times sufficient for diagnosis of acute stroke from computerized tomography (CT) brain scans viewed using an iOS device.

## Methods

One hundred and twenty recent consecutive noncontrast computed tomography (NCCT) brain scans and 70 computed tomography angiogram (CTA) head scans were obtained from the Calgary Stroke Program database. The Calgary Stroke Program is recognized as one of the leading programs in North America for stroke treatment and research. 

### NCCT Brain Scans

NCCT brain scans were obtained using sequential acquisition of data on a 64-row multidetector CT (Somatom Sensation 64, Siemens Healthcare, Germany) from the foramen magnum to the skull vertex using a 5-mm slice thickness. NCCT brain scans were evaluated for early signs of infarction, which includes early parenchymal ischemic changes and dense vessel sign, and to exclude acute intraparenchymal hemorrhage and any stroke mimics, such as tumor, infective/inflammatory disease, or any vascular malformation. Acute parenchymal ischemic changes were graded according to the Alberta Stroke Program Early CT Score (ASPECTS) scoring system [6]. ASPECTS is a 10-point scale that grades the extent of ischemic change within the territory of the middle cerebral artery.

### CTA Head Scans

CTA scans of the head and neck were performed on 64-row multidetector CT (Somatom Sensation 64, Siemens Healthcare, Germany). Data were acquired from the ascending aorta to the skull vertex using a standard spiral acquisition after infusion of 100 ml of nonionic iodinated contrast. Axial slices of 1-mm thickness were obtained from the aortic arch to the skull base, while 0.6-mm axial slices were obtained from the skull base to the skull vertex. All images had a 220-mm field of view and contained 512 x 512 pixels. Reformations with 3-mm slices were performed in the axial, sagittal, and coronal planes for the head region and reviewed on a workstation. For the purpose of this study, only axial CTA head scans were used for interpretation on the iOS device. CTA head scans were evaluated for any intracranial vessel occlusion.

The scans were read by two neuroradiologists. One reader (author PS) interpreted scans first on a workstation located in a radiology reading room equipped with a medical-grade display (hereafter, referred to as the workstation) and then on the iOS device. The second reader (author JM) interpreted scans only on the iOS device. To avoid bias, a delay of 2 weeks was allowed between interpretation on the workstation and interpretation on the iOS device. The readers were blinded to the patients’ clinical data. Furthermore, the patient exams were presented in different orders on the workstation and on the client-server teleradiology system.

The workstation (IMPAX 6.3.1.3815, Agfa Healthcare, Belgium) was connected to a medical-grade 21-inch liquid crystal display (MD21GS-3MP, NEC). This display has a resolution of 2048 x 1536 pixels (pixel pitch = 0.21 mm) and a luminance of 400 (candela) cd/m^2^. All interpretations on the medical workstation were performed in a lighting-controlled (darkened) radiology reading room. All patient imaging exams were then anonymized and loaded on the server to be analyzed on the iOS device. The iOS device used in this study had a 3.5-inch diagonal screen having a resolution of 320 x 480 pixels (pixel pitch = 0.15 mm) and a luminance = 500 cd/m^2^. All interpretations on the iOS device were performed under normal office lighting conditions in a room with overhead florescent light panels.

The time to interpret each exam was recorded by the readers themselves using a digital stopwatch. When using the iOS device, the interpretation time included the time required to launch the application, establish a connection to the server, select the study for interpretation, and perform the interpretation. When using the workstation, the recorded time included the time to select the study and perform the interpretation.

### The Server Configuration

The visualization server had a 2.4 gigahertz (GHz) Intel Core 2 Quad Core central processing unit, 8 GB RAM and two NVIDIA GeForce 8800 (512 MB) graphics cards. It ran Red Hat Enterprise Linux 5 (Red Hat Inc, Raleigh, NC) and included the application ResolutionMD Enterprise (Calgary Scientific Inc, Calgary, Canada). ResolutionMD Enterprise (hereafter referred to as the server software) allows interactive 2-D and 3-D visualization of DICOM images on remote displays via secure hypertext transfer protocol (http). Visualization is initiated by a remote user, who connects to a particular uniform resource locator (URL) address on the server using a Web browser. The server will perform different actions depending upon the URL specified. For example, the visualization server might send a small Flash (Adobe Systems Inc, San Jose, California) or Silverlight (Microsoft Inc, Redmond, Washington) client program to the Web browser. This client program is executed by the browser and used to implement the remote user interface and manage communication with the server.

Initially, the user interacts with the client program to choose a DICOM series to visualize. Typically, the DICOM images are resident on a picture archiving and communication system (PACS). The server then loads the series of 2-D DICOM images from the PACS into memory and reformats them into a 3-D volume. It then performs a rendering operation on the 3-D volume to produce a 2-D frame, which is compressed using a lossy joint photographic experts group (JPEG) algorithm and encoded for transmission to the remote client for display. The JPEG standard includes a user definable “quality factor” that varies between 1 and 100, where 100 is the highest possible lossy JPEG encoding quality. In our study, the quality factor was set to 25 during interactive image presentation. During static image presentation, the quality factor was automatically set to 100. The quality factor has a variable effect on the achievable compression ratio for neurological CT images [7]. Compression ratios were not measured in our studies. However, previous research indicates that for neurological CT scans, a quality factor of 85 produces a compression ratio of 10:1, while a quality factor of 92 produces a compression ratio of 8:1 [7]. These compression ratios are well within published guidelines that suggest a maximum compression ratio of 12:1 for (static) neurological CT scans [8].

All quantitative operations (for example, adjusting the image intensity window and level) are performed by the server on the original DICOM data. The results are then transmitted interactively to the remote client, either as a new encoded frame, or as updated textual information. DICOM files are not transmitted to, or stored on, the client. Nevertheless, the server-side rendered image is stored temporarily in client-side volatile random-access memory (RAM) in both JPEG and decompressed image formats. Any confidential patient information (eg, patient name) that has been rendered into the image pixels is thus temporarily present on the client. Some textual attributes from the originating image’s DICOM file may also be temporarily stored in volatile RAM on the client. These attributes may be used as navigational and informational displays within the client user interface. When the user exits the Web browser or moves to a new webpage, the connection to the server is closed, the rendering operation ends, a blank image is displayed on the client, and the volatile RAM on the client is cleared.

The server software provides a number of visualization protocols, including 2-D, 2-D side-by-side, linear and curvilinear multiplanar reformatting, and several 3-D volume rendering modes. At this level, the system has similarities with others that have been described in the literature [9]. However, unlike previous systems, our server software includes two important novel and proprietary enhancements. The first of these allows more efficient use of available bandwidth. The server software decomposes the sequence of 2-D frames into static and dynamic components. To do this, the server keeps track of the last image sent to the client before compression. When the next image is to be sent, the server compares 8 x 8 blocks within the new image with corresponding blocks of the previous image. Only blocks that contain changes in pixel data between the two images are compressed and transmitted to the client. The second enhancement allows Web-client applications with sophisticated and complex user interfaces. The system provides server- and client-side application program interfaces (APIs) and libraries for maintaining and synchronizing a hierarchical state model between the server and one or more clients, without requiring tight coupling of compatible data types, data structures, and programming languages. Efficiency is achieved by ensuring that the internal state model can be mapped onto the extended markup language (XML), which can be easily manipulated such that only the differences in the state between a client and server are ever transmitted. This allows the application state to be managed efficiently and shared and synchronized simultaneously with multiple clients. In turn, this permits simultaneous collaboration between multiple users across large distances and diverse networks.

This framework also allows a single client application to connect to multiple servers, then observe and manipulate the images and states of those servers. In turn, this allows one to “mix and match” components of multiple server-side applications into a single client-side application.

### The Client Configuration

The iOS device (iPod or iPhone) used in our experiments included 8 gigabytes (GB) of flash memory and an operating system version 3.1.2. The Safari Web browser application available on iOS devices is not capable of running Flash or Silverlight programs. Therefore, we developed a custom application for iOS devices to implement a remote user interface and manage communication with the server. This software was written in Objective-C 2.0 using the Xcode 3.0 integrated development environment. Our application was installed on the iOS device used in this experiment using Apple Inc’s ad-hoc distribution method. This process allows one to install and test applications without having them released on Apple Inc’s online iOS device application store. The version of our iOS device application used in these experiments only exposed some of the advanced visualization modes available in the server software. It allowed 2-D and 3-D visualizations, interactive window/level, translation, rotation, and zoom. In 3-D mode, the user could also select from a range of tissue rendering settings.

**Figure 1:**
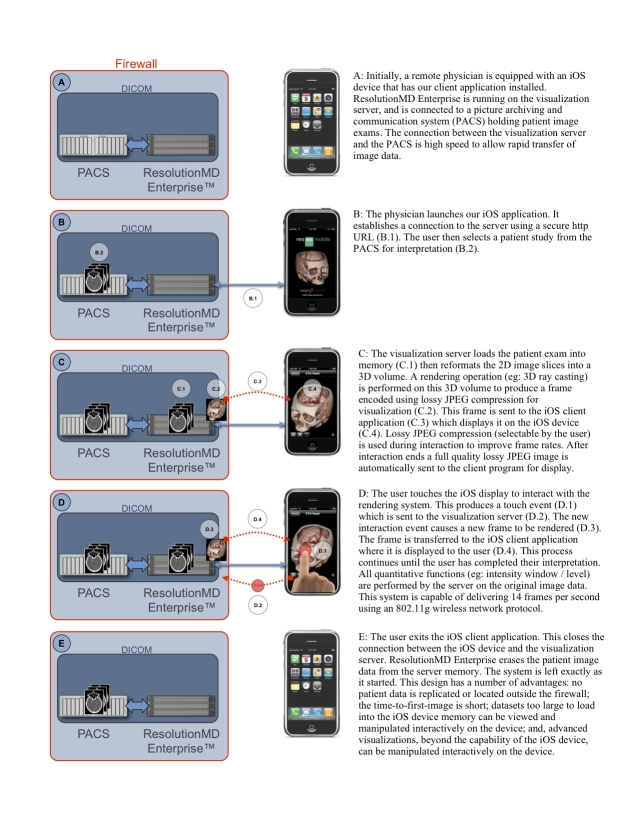
A schematic overview of the client-server teleradiology system

Once launched, the custom iOS device application initiates communication with a remote server running the server software (Figure 1). Users can use default server addresses provided with the iOS device application, or they can enter and save custom server addresses. The iOS device application then captures user interaction events, communicates these to the server, and manages the sequence of encoded frames for visualization. We set the default server address to point to the server used in our experiments. This eliminated the need for the user to specify an address, thereby reducing the delay before image interpretation. Communication between the visualization server and the iOS device occurred over a secure wireless network (Wi-Fi 802.11g). Communication via https to the server over third generation (3G) cellular networks is also possible on the iOS device 3GS. However, preliminary experimentation suggested that frame rates over 3G cellular networks are insufficient for practical use at this time. Consequently, cellular networks were not tested during this study. An overview of the iOS device system is provided in Figure 1.

### Analysis

The interpretations on an iOS device were compared to those of a workstation. Readings of reader 1 (author PS) on the workstation were considered ground truth for comparison. Any differences between the iOS device and workstation platforms in detecting stroke mimics or acute intraparenchymal hemorrhage, acute parenchymal ischemic changes, hyperdense vessel sign on NCCT brain scans and intracranial vessel occlusion on CTA brain scans were considered errors. If the difference in APSECTS scoring was more than one, it was considered a discrepancy in reading. Interrater agreement between the readers was assessed by calculating kappa using Stata 10.0 (StataCorp, College Station, Texas, USA). True positives, true negatives, false positives, and false negatives were obtained, and sensitivity, specificity, and accuracy of detecting the abnormalities on the iOS device were computed. The mean interpretation times on the workstation and iOS device were compared.

## Results

### NCCT Brain Evaluation

Of the 120 NCCT brain scans, poor image quality (from patient motion during scanning) resulted in 8 patient scans being excluded leaving 112 scans for review. There were no stroke mimics identified on the workstation or the iOS device. Thus, none were falsely diagnosed as stroke on the iOS device.

Acute intracranial hemorrhage (ICH) was detected using the workstation in 7 of the 112 patients. The remaining 105 patients were then assessed for early ischemic changes. Both readers correctly diagnosed intracranial hemorrhage in all 7 patients. The sensitivity, specificity, and accuracy of detecting hemorrhage on NCCT brain scans were 100% using the iOS device with perfect interrater agreement (kappa = 1).

**Figure 2:**
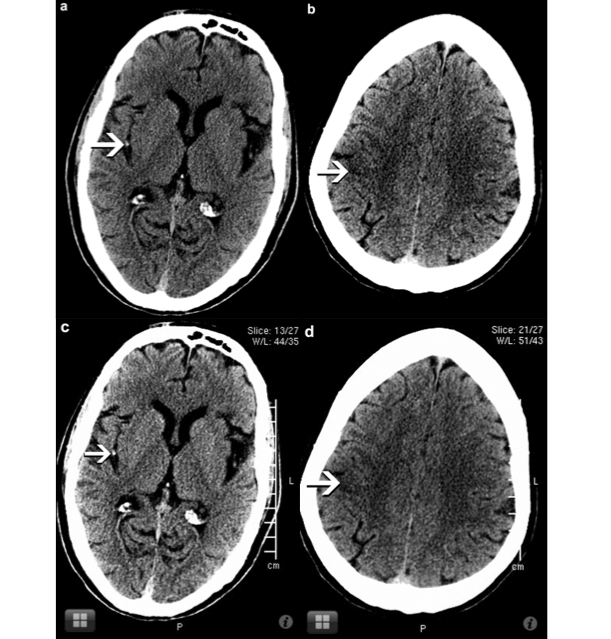
In image *a*, the workstation monitor images show a dense MCA sign (arrow) and in image *b*, acute ischemic change in the ASPECTS M6 region (arrow). Corresponding iOS device images show a dense MCA sign (arrow) in image *c* and acute ischemic change in ASPECTS M6 region (arrow) in image *d*.

Acute parenchymal ischemic changes were seen when using the workstation in 34 of 105 patients. The sensitivity, specificity, and accuracy of detecting acute parenchymal ischemic change (Figure 2) were 94.1%, 100%, and 98.09% respectively for reader 1 and 97.05%, 100%, and 99.04% for reader 2 using the iOS device. There was nearly perfect interrater agreement (kappa = .8) between the readers.

**Figure 3:**
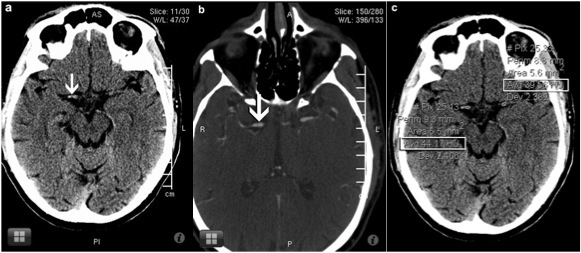
On an iOS device, an NCCT brain scan shown in image *a* was incorrectly interpreted as having a dense MCA sign (arrow). On an iOS device, a CT angiography brain scan (image *b*) demonstrates a normal patent vessel. On the workstation monitor, an NCCT brain image *c* shows nearly the same Hounsfield units (open rectangles) of both vessel segments, thus avoiding the error made on the iOS device.

Dense vessel sign was detected in 18 of 105 patients with acute ischemic changes seen on the workstation. There were 4 false positives by reader 1, and 5 false negatives by reader 2 in diagnosing dense vessel sign on the iOS device. The sensitivity, specificity, and accuracy of detecting dense vessel sign (Figures 2 and 3) on NCCT brain scan were 100%, 95.4%, and 96.19% respectively for reader 1 and 72.2%, 100%, and 95.23% for reader 2 using the iOS device. There was good interrater agreement (kappa = .69) between the readers.

The mean time to interpret NCCT exams on the workstation was 2.1 minutes (SD 0.77), while the mean time using the iOS device was 2.7 minutes (SD 0.9) for reader 1 and 2.3 minutes (SD 1.4) for reader 2. These mean times were not statistically significantly different from each other at *P* > .05. The interpretation time ranged between 1 and 6 minutes on both platforms.

### CT Angiogram Evaluation

Of the 70 CTA head scans, patient motion and/or poor contrast opacification of vessels resulted in the exclusion of 5 poor quality CTA exams, leaving 65 exams for our review. Vessel occlusion was detected in 18 of the 65 patients on the workstation.

**Figure 4:**
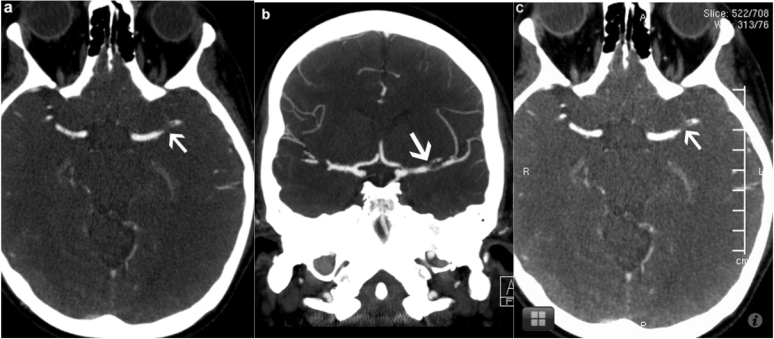
The workstation monitor axial and coronal images *a* and *b* show acute thrombus (arrow) in the proximal segment of the left MCA. Corresponding iOS device axial image *c* shows acute thrombus (arrow) in the proximal segment of the left MCA.

Both readers correctly diagnosed vessel occlusion (Figure 4) in 17 of 18 patients on the iOS device. However, both readers missed an occlusion of the V4 segment of the vertebral artery using our system. All patients with patent intracranial vessels were correctly diagnosed using our system without any false-positive errors.

The sensitivity, specificity, and accuracy of detecting vessel occlusion on CT angiography scans were 94.4%, 100%, and 98.46% respectively for both readers using the iOS device. There was perfect interrater agreement (kappa = 1) between the readers.

The mean time to interpret CTA exams on the workstation was 3.5 minutes (SD 1.2), while using the iOS device the mean time was 3.63 minutes (SD 1.48) for reader 1 and 3.83 minutes (SD 0.83) for reader 2. These mean times were not statistically significantly different from each other at *P* > .05. The interpretation time ranged between 1 and 7 minutes on both platforms.

True positives, true negatives, false positives, and false negatives and sensitivity, specificity, and accuracy of detecting the abnormalities on the iOS device by both readers are shown in Tables 1 and 2.

**Table 1:** Sensitivity, specificity, and accuracy of stroke diagnosis using the client-server teleradiology system by reader 1

	Work-station (reader 1)	iOS Device (reader 1)	TP^a^	FP^b^	TN^c^	FN^d^	Sensitivity	Specificity	Accuracy
Hemorrhage	7/112	7/112	7	0	105	0	100%	100%	100%
Acute parenchymal change	34/105	32/105	32	0	71	2	94.11%	100%	98.09%
Dense vessel sign	18/105	22/105	18	4	83	0	100%	95.4%	96.19%
Vessel occlusion on CTA	18/65	17/65	17	0	47	1	94.4%	100%	98.46%

^a^True positive on the iOS device

^b^False positive on the iOS device

^c^True negative on the iOS device

^d^False negative on the iOS device

**Table 2:** Sensitivity, specificity, and accuracy of stroke diagnosis using the client-server teleradiology system by reader 2

	Work-Station ( reader 1)	iOS device (reader 2)	TP^a^	FP^b^	TN^c^	FN^d^	Sensitivity	Specificity	Accuracy
Hemorrhage	7/112	7/112	7	0	105	0	100%	100%	100%
Acute parenchymal change	34/105	33/05	33	0	71	1	97.05%	100%	99.04%
Dense vessel sign	18/105	13/105	13	0	87	5	72.2%	100%	95.23%
Vessel occlusion on CTA	18/65	17/65	17	0	47	1	94.4%	100%	98.46%

^a^True positive on the iOS device

^b^False positive on the iOS device

^c^True negative on the iOS device

^d^False negative on the iOS device

## Discussion

Acute ischemic stroke is the most common form of stroke, and it is also the most treatable. For each minute of acute brain ischemia, 1.9 million neurons are destroyed [10]. Treatment with thrombolysis is highly time dependent and entirely dependent upon quick imaging to make an inclusive diagnosis. Equally, expertise remains limited. Any method that can reduce the time from image acquisition to expert review and decision is beneficial. It is clear that every 15-minute delay results in a measureable reduction in the probability of good outcome after thrombolysis [11]. Hence, rapid image visualization by an expert wherever that expert is is a key factor in improving patient outcomes.

We developed a teleradiology system based on a client-server architecture that enables rapid access to radiological images on a current generation smartphone device. Through this system, a physician can securely assess remote imaging wherever a cellular or wireless network is available, which allows urgent management decisions to be made.

It is important to exclude any intracranial hemorrhage, as this is a contraindication to thrombolytic agents. A previous study has shown that acute intracranial hemorrhage is detected with high accuracy and interrater reliability (kappa = .87 - .94) on NCCT brain scans [12]. In our study, the presence of intraparenchymal hemorrhage was accurately diagnosed with perfect interrater reliability on NCCT brain scans reviewed on an iOS device. Our experience reflects that of Toomey et al [13], who found a personal digital assistant to be accurate in diagnosing acute intraparenchymal hemorrhage when compared with a workstation.

The extent of early ischemic changes in the parenchyma has been correlated with poorer clinical outcomes and increased risk of hemorrhage [14]. Previous meta-analysis has shown mean sensitivity of 66% (range 20% - 87%) and mean specificity of 87% (range 56% - 100%) with varying interrater agreement (kappa = .14 - .78) to detect an early infarction sign on NCCT brain scans [15]. Studies have been published demonstrating good sensitivity and specificity in detecting early ischemic signs using the ASPECTS scoring system [16,17]. ASPECTS is a well-validated scoring system that has good interrater reliability (kappa .71 - .89) [6,16]. In our study, there was high accuracy with nearly perfect interrater agreement (kappa = .8) in detection of early ischemic parenchymal changes on an iOS device. There were no false-positive diagnoses of early ischemic parenchymal changes by either of the readers using our system. Importantly, no large infarcts were missed by either of the readers using our system.

Hyperdense vessel sign on NCCT brain scan is a marker of intraluminal thrombus [18,19]. Studies have shown that hyperdense vessel sign is a highly specific but only moderately sensitive indicator of thromboembolic occlusion [18] with a wide range of interrater reliability (kappa = .36 - 1.00) [15]. False-positive hyperdense sign can be seen in calcified atherosclerotic vessel or a high hematocrit [20]. In this study, there were discrepancies in the detection of dense vessel signs on the iOS and the workstation with average interrater agreement (kappa = .69). There were 4 false positives by reader 1, and 5 false negatives by reader 2 in detecting hyperdense vessel sign on the iOS device. We feel that measuring the density in Hounsfield units and comparing with the opposite side vessel helps reduce false-positive and false-negative diagnoses rather than relying solely on the observed local CT image contrast. The workstation software allowed the reader to analyze and display the CT scan Hounsfield units (Figure 3). The server software does provide functions to analyze Hounsfield units. However, the iOS client software used in this study did not expose this functionality to the user. It was subsequently added to the version licensed by Health Canada.

Diseases that can mimic stroke such as tumors, infection/inflammatory diseases, and functional conditions may be sometimes difficult to distinguish from acute stroke based solely on neurological examinations. In addition, NCCT brain scans may be normal in the presence of acute ischemia if the patient is imaged very early. Thus, an objective method for confirming intracranial vessel occlusions prior to treatment is preferable. CT angiography is rapid and widely available. The location and extent of intracranial thrombus has been shown to predict functional outcome and risk of parenchymal hematoma [21]. CTA and perfusion studies provides an effective add-on to NCCT brain scans in acute stroke imaging by significantly increasing the sensitivity and reliability of acute infarct and vessel occlusion detection [22]. In our study, there was high sensitivity, specificity, and accuracy and good interrater agreement to diagnose intracranial occlusion on CTA scans reviewed on an iOS device. One patient with an occlusion of the distal segment of the vertebral artery was identified as normal on an iOS device. This false-negative error may have been avoided if our system allowed the user to view orthogonal and multiplanar reformats of the source data. Once again, the server software does provide functions for orthogonal and multiplanar reformatting. However, the iOS client software used in this study did not expose this functionality to the user. These functions were added to the new version licensed by Health Canada.

Prior to developing our iOS client software, we had a number of concerns about both the iOS device and the client-server architecture that we thought might limit clinical utility. In particular, the iOS device display is (1) much smaller than that of a workstation; (2) has only 320 x 480 pixels—insufficient to display a full 512 x 512 CT scan image; and (3) is not a medical-grade display being used in a lighting-controlled radiology reading room. In addition, we were concerned that our client-server architecture might provide insufficient interactivity for practical use. In practice, we discovered several factors that helped alleviate our concerns. First, in most NCCT brain scans, the patient’s brain does not fill the entire 512 x 512 image array. In addition, readers were able to easily observe all image regions at native resolution through interactive translation, while interactive zoom allowed them to focus in on image areas of interest. Next, when interpreting images, users tend to position the iOS device display much closer to their eyes, which helps compensate, to some degree, for the smaller size of the iOS device display. In addition, the iOS device display has 25% higher pixel density and luminance than those on the medical-grade liquid crystal display (LCD) monitor of the workstation used in these experiments.

There were limitations in our study. In particular, it was a retrospective analysis performed of 173 patients’ brain scans by two neuroradiologists in a research laboratory environment with high-speed network infrastructure. Only 7 of 112 (6%) hemorrhagic strokes occurred among these patients, which would cause the sensitivity of hemorrhagic diagnosis to be high regardless of how the images were interpreted. Consequently, care should be taken when drawing conclusions from our results. In the future, a larger prospective study performed by physicians on-call working under clinical constraints will be required to demonstrate the clinical utility of our system. We would also like to test the potential usefulness of our system for other acute conditions like renal colic, skeletal trauma, and acute coronary disease.

The system should provide practical frame rates over cellular or wireless networks. In our experience, a single visualization server can accommodate 10 or more simultaneous iOS device users and is capable of delivering and displaying up to 14 frames per second on an iOS device connected over a 802.11g Wi-Fi network. The frame rate was enough to provide sufficient interactivity for comfortable use. However, the frame rate on a 3G cellular network was 1 to 4 frames per second, which was insufficient for practical use. We know that fourth generation (4G) cellular networks are now installed in many metropolitan centers. We estimate that the higher bandwidth of these new cellular networks should allow 10 to 15 frames per second to be delivered to smartphones. However, currently only the iPhone 4 and a few Android-based smartphones are capable of utilizing greater network bandwidth.

Our system has been approved as a medical device under device class 2 with Health Canada. It is under review by the US Food and Drug Administration. We suspect that the regulatory agencies will have two major concerns regarding this system: that it has functional equivalence to previously licensed image interpretation platforms and that it does not unduly jeopardize patient confidentiality. Our client-server architecture may help address each of these concerns. First, protection of patient confidentiality is aided by the fact the no DICOM data is stored outside the hospital firewall on the iOS device. Second, since the server performs all rendering operations, it may be possible to expose sufficient workstation-class functionality on the iOS device to demonstrate functional equivalence. For example, the iOS device and other current generation smartphones do not have sufficient computational resources and rapid access memories to perform interactive multiplanar reformatting or advanced 3-D visualization. Yet, these functions may be required by the regulatory agencies to demonstrate functional equivalence to existing interpretation platforms. We are investigating new methods to usefully expose additional advanced visualization capabilities to the remote user on an iOS device and to optimize both server and client performance to enhance interactivity over both Wi-Fi and cellular networks.

In summary, the smartphone client-server teleradiology system appears promising and may have the potential to allow urgent management decisions in acute stroke. However, this study was retrospective, involved relatively few patient studies, and only two readers. Widespread conclusions about its clinical utility, especially in other diagnostic use cases, should not be made until additional studies are performed.

## Abbreviations

3G: third generation
4G: fourth generation
API: application program interfaces
ASPECTS: Alberta Stroke Program Early CT Score
CT: computerized tomography
CSI: Calgary Scientific Inc
CTA: computed tomography angiogram
DICOM: digital imaging and communications in medicine
GB: gigabytes
GHz: gigahertz
http: hypertext transfer protocol
ICH: intracranial hemorrhage
JPEG: joint photographic experts group
LCD: liquid crystal display
MB: megabytes
MCA: middle cerebral artery
NCCT: noncontrast computed tomography
PACS: picture archiving and communication system
RAM: random-access memory
URL: uniform resource locator
XML: extended markup language
